# Preparation, Characterization, and In Vivo Evaluation of Gentiopicroside-Phospholipid Complex (GTP-PC) and Its Self-Nanoemulsion Drug Delivery System (GTP-PC-SNEDDS)

**DOI:** 10.3390/ph16010099

**Published:** 2023-01-09

**Authors:** Yingpeng Tong, Wen Shi, Qin Zhang, Jianxin Wang

**Affiliations:** 1 Institute of Natural Medicine and Health Product, School of Advanced Study, Taizhou University, Taizhou 318000, China; 2 Department of Pharmaceutics, School of Pharmacy, Fudan University, Key Laboratory of Smart Drug Delivery, Ministry of Education, Shanghai 201203, China; 3 Institute of Integrative Medicine, Fudan University, Shanghai 201203, China

**Keywords:** gentiopicroside, phospholipid complex, self-nanoemulsion drug delivery system, oral bioavailability, pharmacokinetics

## Abstract

The objective of the present study was to develop a gentiopicroside-phospholipid complex (GTP-PC) and its self-nanoemulsion drug delivery system (GTP-PC-SNEDDS) to increase the oral bioavailability of gentiopicroside (GTP). The factors affecting the formation of GTP-PC were studied with the complexation efficiency and dissociation rate. The properties of the complex were investigated by means of differential scanning calorimetry (DSC), X-ray diffraction (XRD), Fourier transform infrared spectra (FT-IR), dissolution, etc. Then, GTP-PC was loaded into SNEDDS by investigating the effects of weight ratios of GTP-PC to blank SNEDDS, preparation technology, dilution media, and dilution multi, based on the screening results of oils, surfactants, and cosurfactants. In rats, GTP, GTP-PC, and GTP-PC-SNEDDS were orally administered at different times, and GTP concentrations were determined using RP-HPLC. The optimal GTP-PC was prepared with tetrahydrofuran as the reaction solvent, GTP:phospholipid = 1:2, and stirring for 4 h. The optimal prescription for GTP-PC-SNEDDS was as follows: Maisin 35-1:Miglycol = 30%, Labrasol:Cremophor EL = 1:4 = 40%, Transcutol P = 30%; Maisin 35-1:Miglycol = 12, and the ratio of GTP-PC to blank was 1:10—then the mixture was stirred at 37 °C for 1 d and then placed for 2 d to form stable GTP-PC-SNEDDS. After oral administration of GTP, GTP-PC and GTP-PC-SNEDDS, and mean plasma GTP concentration–time curves were all in accordance with the single-compartment model. The C_max_, AUC_0–∞_, and Fr of the three formulations were significantly higher than that of GTP, demonstrating that GTP was metabolized rapidly, and its higher bioavailability could be achieved by the formation of GTP-PC and GTP-PC-SNEDDS. Among the three formations, the bioavailability of GTP-PC-SNEDDS was highest, with approximately 2.6-fold and 1.3-fold of Fr value, compared with GTP-PC (suspension) and GTP-PC (oil solution), respectively. Compared with GTP, GTP-PC and GTP-PC-SNEDDS enhanced the bioavailability of GTP significantly. In the future, this study could serve as a reference for clinical trials using GTP-PC and GTP-PC-SNEDDS.

## 1. Introduction

**Figure 1:**
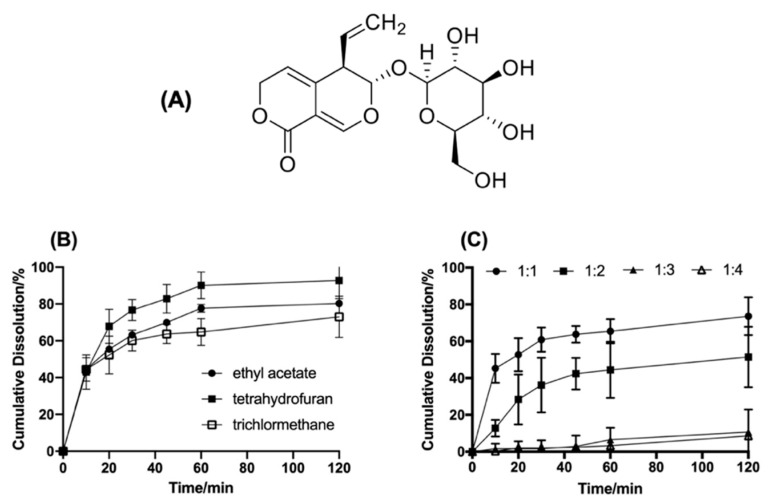
The structure of GTP and its influencing factors on the formation of phospholipid complexes. (**A**): The structure of GTP. (**B**): Cumulative Dissolution of phytosome of different type of solvent in phosphate buffer saline (pH 6.8). (**C**): Cumulative Dissolution of phytosome of different molar ratios of GTP and phospholipids.

Gentiopicroside (GTP, Figure 1A), a kind of iridoid glycoside, is one of the key active components from Gentiana species [1,2,3]. It exhibits many activities, including anti-inflammatory, analgesic, antioxidant, and anti-hepatotoxic activities, which imply its benefit in the treatment of rheumatoid arthritis, liver illness (hepatitis), fever, digestive, intestinal disorders, and so on [4,5,6,7,8,9].

Unfortunately, the clinical efficacy was limited by the low bioavailability of oral GTP. Several studies attributed the low bioavailability of oral GTP to the first-pass metabolism, bacterial metabolic processes, or decomposition in the intestine, as well as to poor absorption from the gastrointestinal tract [10,11,12,13,14,15,16]. To overcome the problem of the slight clinical efficacy of GTP, it is important to improve its bioavailability. Some reports had exposed that the bioavailability of GTP could be significantly improved by the interaction of the compound–compound in an herb extract [11] or the herb–herb in a formulae decoction [12,16]. Additionally, no data about pharmaceutical approaches have been published, in relation to improving the bioavailability of GTP after oral administration.

The formation of a drug–phospholipid complex is an important, and the most common, way to improve bioavailability. Up until now, several drug–phospholipid complexes have been approved to use in clinical treatment, such as Meriva^®^ (curcumin-PC) [17], Siliphos^®^ (Silybin-PC) [17]. SNEDDS, which consists of a drug, oil, surfactant, and co-surfactant, can produce an oil-in-water (O/W) nanoemulsion with a droplet size of less than 100 nm by gently mixing with water [18,19,20,21]. It is another pharmaceutical means to enhance the drug’s bioavailability. More importantly, the combination of PC and SNEDDS can overcome the same limitations of drugs with low oral bioavailability. It has been proposed that combining PC with SNEDDS can enhance the oral bioavailability of bioactive compounds or biomacromolecules, such as morin [22,23,24,25], akebia saponin D [26,27], rosuvastatin calcium [28], paclitaxel [29], curcumin [30], ellagic acid [31], baicalin [32], and matrine [33].

In the above experiments, the tested compounds are water insoluble and belong to classes 2 or classes 4 of the biopharmaceutical classification system. However, there are no related studies on class 3 compounds. Classified as class 3 drugs, they are highly soluble in gastrointestinal fluids, but possess low absorption membrane permeability, often resulting in low bioavailability [34]. GTP was a typical class 3 compound with high water solubility (7.65 g/100 g at 23 °C) and low membrane permeability [35]. So, GTP was adopted in this study, and then GTP-PC and GTP-PC-SNEDDS were optimized to test their advantages on enhancing GTP’s oral bioavailability.

## 2. Results and Discussion

### 2.1. Factors Affecting the Formation of GTP-Phospholipid Complex

#### 2.1.1. Different Type of Phospholipids

The complexation efficiency and dissociation rate of GTP-PC of different type of phospholipids, including hydrogenated phospholipids, soybean phospholipids, and egg yolk phospholipids, are shown in Table 1. The results display that the complexation efficiency of the GTP-PC prepared by the soybean phospholipid and egg yolk phospholipid exceeded 95%. The dissociation rate of GTP-PC in pH 6.8 PBS was hydrogenated phospholipid > egg yolk phospholipid > soybean phospholipid, indicating that the stability of hydrogenated phospholipids prepared by hydrogenated phospholipid was poor, and it was easy to dissociate in aqueous environment. The cost of egg yolk phospholipid is higher than that of soybean phospholipid. Considering the complexation efficiency, dissociation rate, and cost factors, soybean phospholipid was selected for preparing GTP-PC.

#### 2.1.2. Dissolving Solvents

The solubility of GTP and GTP-PC is shown in Table 2. The results show that GTP is insoluble in dichloromethane, ethyl acetate, and tetrahydrofuran, but GTP-PC and soybean phospholipid are easily soluble. In this case, the formation of GTP-PC could be judged according to the change of solubility. Then, the most suitable solvent was chosen further by complexation efficiency and dissociation. The results are shown in Table 3 and Figure 1B, indicating that the phospholipid complex prepared in tetrahydrofuran had the highest complexation efficiency and slowest dissociation. Therefore, tetrahydrofuran was identified as the dissolving solvent.

#### 2.1.3. Molar Ratio of GTP to Phospholipid

Complexation efficiency and dissociation of the GTP-PC of different molar ratios of GTP and phospholipid are shown in Table 4 and Figure 1C. The data indicated that the complexation efficiency of phospholipid complex was close to 100% for the GTP of GTP:phospholipid ≤ 1:1. The dissociation rate increased with the ratio of GTP-to-phospholipid increase, suggesting that the greater the amount of phospholipid, the higher the stability of GTP-PC. However, the content of phospholipid could not be too much, due to the poor dispersion and fluidity of the GTP-PC of the drug-to-lipid ratio ≥ 1:3. Additionally, according to the results of the bioavailability, the molar ratio of GTP and phospholipid was identified as 1:2 [36].

#### 2.1.4. Stirring Time

It was found that, with the stirring time increasing from 0.5 to 4 h, the complexation efficiency increased from 83.57% to 99.50%, and at 4 h reached the peak. When stirring for 5 h and 6 h, the complexation efficiency declined to 92.63% and 88.63%. This may be due to the instability of GTP. Therefore, the optimal stirring time was 4 h.

### 2.2. Characterization of GTP-PC

#### 2.2.1. Differential Scanning Calorimetry (DSC)

**Figure 2:**
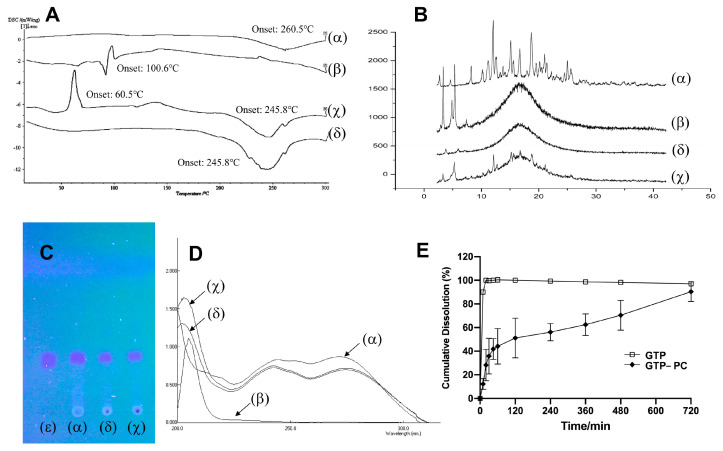
Characterization of GTP-PC. (**A**): Differential scanning calorimetry (DSC). (**B**): X-ray diffractometry (XRD). (**C**): Thin-layer chromatography (TLC). (**D**): Ultraviolet (UV) spectra. (**E**): Degradation of GTP-PC in phosphate buffer solutions and diluted hydrochloric acid at different pH (n = 3). GTP (α), phospholipids (β), physical mixture (χ), GTP-PC (δ), and standard GTP (ε).

In DSC, an interaction can be detected by eliminating endothermic peaks, showing new peaks, changing peak shape, and changing the peak temperature and relative peak area or enthalpy [37]. The DSC curves of the GTP, phospholipids, physical mixture, and GTP-PC are shown in Figure 2A. The GTP displayed an abroad endothermal peak at 260.5 °C (Figure 2Aα). The phospholipid had a peak at 100.6 °C (Figure 2Aβ). The DSC of the phospholipid complex showed that the phospholipid peak vanished, and the onset temperature was lower than GTP’s, which was at 245.08 °C (Figure 2Aδ). The GTP and phospholipids were thought to interact through hydrogen bonds or van der Waals forces. As the GTP and phospholipid polarity parts were combined, the carbon–hydrogen chain of phospholipids was free to turn and enclose the polarity parts and GTP of the phospholipids. After that, the sequence between the aliphatic hydrocarbon chains of phospholipids decreased and the peak of phospholipids disappeared, lowering the onset temperature of GTP [38]. The physical mixture of the GTP and phospholipid showed two peaks. The former was 60.5 °C, lower than the onset temperature of phospholipid; the other was 245.8 °C, the same with the onset temperature of GTP-PC (Figure 2Aχ). It was thought that, as the temperature increased, the phospholipid melted, and the drugs dissolved into it, partially forming phospholipid complexes.

#### 2.2.2. X-ray Diffractometry (XRD)

The powder X-ray diffraction patterns of the GTP, phospholipids, physical mixture, and complex are shown in Figure 2B. The GTP displayed sharp crystalline peaks. In contrast, phospholipids were amorphous and lacking crystalline peaks. Despite the physical mixtures of the GTP and phospholipid, some drug signals could still be detected. However, the crystalline peaks had disappeared in the GTP-PC. The combination of the polar ends of phospholipid with GTP led to their being highly dispersed, thus inhibiting their crystalline characteristics.

#### 2.2.3. Fourier Transform Infrared Spectra (FT-IR)

**Figure 3:**
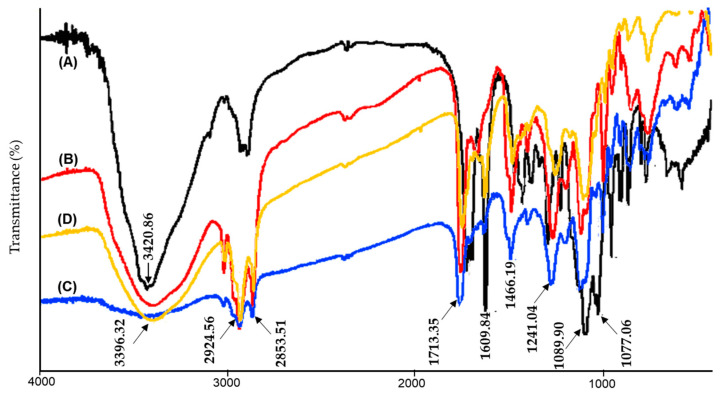
Fourier Transform Infrared spectra (FT-IR) of GTP-PC. GTP (**A**), phospholipids (**B**), physical mixture (**C**), GTP-PC (**D**).

The FT-IR of the GTP, phospholipids, physical mixture, and GTP-PC are shown in Figure 3A–D, respectively. The FT-IR spectrum of GTP was shown in Figure 3A, disclosing a broad band of 3600–3000 cm^−1^ for hydrogen-bonded O-H stretching vibrations. The absorption bands at 1713.35, 1609.84 cm^−1^ related to the C=O and C=C stretching. The signal at 1273.48 and 1007.06 cm^−1^ were assigned to C–O–C vibration. This FT-IR spectrum was consistent with the previously published reports. The stretching vibration of hydroxyl in GTP (υOH, 3420.86) and phospholipids (υOH, 3389.75) were superimposed in the physical mixture, forming a large, wide peak. The vibration of the hydrocarbon of saturated long-chain fatty (υCH, 2924.56, 2853.51), carbonyl of fatty acid ester (υC=O, 1737.68), phosphorus oxygen double bond (υP=O, 1241.04), and phosphorus oxygen single bond (υP-O-C, 1089.90) in phospholipid can be found, indicating little interaction between the GTP and phospholipid in the physical mixture (Figure 3C). In the phospholipid (Figure 3B), there were strong hydrogen bonds, so the vibration of hydroxyl was very strong and had a relatively low wavenumber (3389.75). This was due to the fact that the phospholipid phosphate and quaternary ammonium in the phospholipids had a strong ability to ionize, and hydrogen bonds formed by positive and negative ions were significantly strong, possibly with ionic character. However, the combination of phospholipid and GTP made new hydrogen bonds, formed between the two, and also destroyed part of the hydrogen bonds between the phospholipids (Figure 3D), resulting in the vibration of the hydroxyl moving to a higher wavenumber (υOH, 3396.32). Moreover, the υC=O and υP-O-C in phospholipids moved to a lower wavenumber (υC=O, 1737.68 to 1735.78; υP-O-C, 1089.90 to 1085.39), indicating that new hydrogen bonds may have been formed.

The FT-IR of the GTP, phospholipids, physical mixture, and GTP-PC is shown in Figure 3A–D, respectively. The GTP spectrum (Figure 3A) disclosed the characteristic hydrogen-bonded O-H stretching vibration at 3600–3000 cm^−1^, C=O stretching vibration at 1713.35 cm^−1^, C=C stretching vibration at 1609.84 cm^−1^, and C–O stretching vibrations at 1273.48, 1083.96, and 1007.06 cm^−1^. The characteristic C-H stretching and bending vibrations of the long fatty acid chain of PC shown in Figure 3B were at 2924.56, 2853.51, and 1466.19 cm^−1^. Carbonyl of fatty acid ester (υC=O) at 1737.68 cm^−1^, phosphorus oxygen double bond (υP=O) at 1241.04 cm^−1^, and phosphorus oxygen single bond (υP-O-C) at 1089.90 cm^−1^ in phospholipid can also be found. In the spectrum of the physical mixture of GTP and PC, the peaks of O-H stretching vibrations of GTP and PC were superposed to form a wide peak at 3409.70 cm^−1^, with the other main peaks from GTP and PC still existing in the physical mixture of GTP and PC, but the intensity of some peaks from GTP were significantly weakened, such as the C=C stretching vibration at 1609.84 cm^−1^ and C–O stretching vibration at 1007.06 cm^−1^. However, the combination of the phospholipid and GTP may form new hydrogen bonds between them and destroy part of the hydrogen bonds between the phospho-lipids (Figure 3D), resulting in the hydroxyl vibration of PC moving to higher wavenumber at 3396.32 cm^−1^. The intensity of the peaks from GTP at 1609.84 cm^−1^ and 1007.06 cm^−1^ were also weakened, but they were relatively stronger than those from the physical mixture of GTP and PC.

#### 2.2.4. Thin-Layer Chromatography (TLC)

Only one spot with the same Rf as GTP was observed in the chromatogram of GTP-PC, similar to that of the physical mixture (Figure 2C). As the GTP and phospholipid have no chemical groups that can react with each other under our preparation conditions, they were unable to form new compounds. Therefore, the TLC did not reveal any new spots, and the UV confirmed the result.

#### 2.2.5. Ultraviolet (UV) Spectra

As can be seen in Figure 2D, the phospholipid exhibited only end absorptions close to 200 nm, while GTP, the physical mixture, and GTP-PC showed nearly identical absorption curves. All of them exhibited two characteristic absorption bands at 240 and 270 nm. Therefore, the UV spectra of GTP was not changed during the combination with the phospholipid, indicating that no chemical groups could react between the GTP and the phospholipid.

#### 2.2.6. Dissolution Studies in Phosphate Buffer Saline (pH 6.8)

Figure 2E shows the dissolution profile of GTP from the GTP-PC and GTP in phosphate buffer saline (pH 6.8). The dissolution of GTP was complete in 30 min, while the GTP from GTP-PC had a fast dissolution in the 0–2 h (about 50%); then, the dissolution tended to increase slowly in 2–12 h, and eventually, the phospholipid complex almost completely dissolved in 12 h. Therefore, the GTP and GTP-PC had significantly different dissolution characteristics, in that the dissolution of GTP in GTP-PC was delayed, compared with the GTP material.

### 2.3. Preparation Process of GTP-PC-SNEDDS

#### 2.3.1. Screening of Oils, Surfactants and Cosurfactant

Among the oils surfactants and cosurfactants that have been experimentally investigated and shown in Table 5, imwitor 742, labrasol, and transcutol P had the highest solubility, which were 0.146 ± 0.028, 0.107 ± 0.011, and 0.315 ± 0.012 g/g, respectively. Additionally, the mixtures of Maisin 35-1/Miglycol 812N, Imwitor742/Miglycol 812N, and Labrasol/Cremophor EL with different ratios also had a relatively high solubility.

In previous preliminary studies on the blank formulation of SNEDDS, the SNEDDS could not have been formed by choosing Imwitor 742 or maisin 35-1 as the oil, Labrasol as the surfactant, or Transcutol P as the cosurfactant. Only when the oil loading was less than 10% could the microemulsion be infinitely diluted, but layered after 30 min, which might be related to the weak emulsifying ability of Labrasol. It was reported that the combination of Labrasol and Cremophor EL in the formulation could easily form self-microemulsion because of the high emulsifying ability of Cremophor EL and the excellent dissolving capacity of Labrasol. When the ratio of Labrasol:Cremophor EL = 1:4, the microemulsion area was larger. When the surfactant was changed to Labrasol:Cremophor EL = 1:4, the formed SNEDDS would not layer after 24 h, while the oil loading was still less than 10%, which might have been related with the weak polarity of Imwitor 742. However, the mixture of Maisin 35-1:Miglyol 812N and Imwitor742:Miglyol 812N would significantly improve the oil loading of SNEDDS, which is consistent with the previous reports. After the Maisin 35-1 was mixed with Miglyol 812N, the mixture would change the polarity of the oil phase, improve the ability of oil phase molecule penetrating into surfactant, and reduce the interfacial tension between oil and water, which would result in the formulation of stable microemulsion droplets. In the mixture, the combination of the long-chain oils solubilizing in microemulsions and short-chain oils acting as cosurfactants would help the hydrophilic groups of oil molecules to disperse in polar cephalic groups of surfactants form chelating effect, which would increase the formation area of microemulsions.

**Figure 4:**
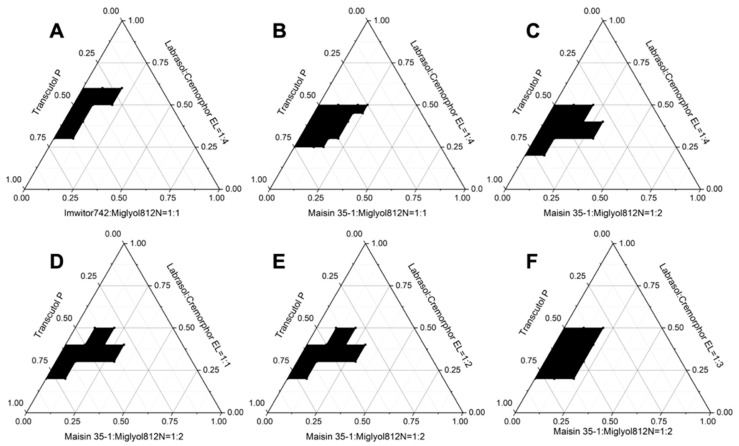
Pseudo-ternary phase diagrams of different compositions of oil, surfactant, and co-surfactant. The region of self-microemulsifaction is in black. (**A**,**B**) indicates effects of different oil on the efficient self-microemulsifacation. The oil phase was as follows: (**A**) as Imwitor742:Miglyol 812N = 1:1; (**B**) as Maisin 35-1:Miglyol 812N = 1:1. (**C**–**F**) indicates effects of different ratios between Labrasol and Cremophor EL from 1:1 to 1:4 on the efficient self-microemulsifacation, respectively.

Based on previous results shown above, the pseudo-ternary phase diagrams were constructed by selecting Imwitor742:Miglyol 812N = 1:1 or Maisin 35-1:Miglyol 812N = 1:1 as the oil phase, Labrasol:Cremophor EL = 1:4 as the surfactant, and Transcutol P as the cosurfactant. It can be seen that the self-microemulsifying region of Maisin 35-1:Miglyol 812N = 1:1 was bigger than that of Imwitor742: Miglyol = 1:1 (Figure 4A,B). The effects of different weight ratios of Maisin 35-1 and Miglyol 812N on the self-microemulsifying region was shown in Figure 4B,C, and Maisin 35-1:Miglyol 812N = 1:2 was chosen as oil phase because of its lager self-microemulsifying region and higher oil loading. Labrasol:Cremophor EL = 1:4 would also been selected by comparing the effects of different weight ratios on the self-microemulsifying region and oil loading, which are presented in Figure 4C–F.

#### 2.3.2. Factors Affecting the Formation of GTP-PC-SNEDDS

In the present work, the effect factors for preparation of GTP-PC-SNEDDS, including the weight ratios of GTP-PC to blank SNEDDS, preparation technology, dilution media, and dilution multi, were further optimized, and the results are presented in Table 6, Table 7, Table 8 and Table 9.

As described in Table 6, the formulation with the weight ratio of 1:10 (GTP-PC:blank SNEDDS) could form a stable self-microemulsion in both the PBS and HCl. This might be related to the solubility of GTP-PC. When the weight ratio was 1:10, the most stable self-microemulsion with the smallest particle size could be formed. With the increase of drug concentration, resulting in the increase of phospholipids, the particle size of self-microemulsion became bigger, which would affect the efficiency of the self-microemulsification. In addition, the undissolved drugs also had some influence on the particle size and stability of the system. Therefore, the ratio of drug-to-blank medium was 1:10 (*w*/*w*).

As shown in Table 7, the GTP-PC-SNEDDS prepared by ultrasonic method and volution would become turbid after dilution, and then be clear after standing for 30 min. Therefore, the excessively vigorous preparation process had a certain influence on the self-microemulsifying drug delivery system. Prepared by placing at different temperature, the self-microemulsifying drug delivery system would become layered, implying that it was an unstable system. The GTP-PC-SNEDDS, prepared by stirring at room temperature, was the most stable system with the smallest particle size and the shortest self-microemulsification time.

The effects of different dilution media on the efficiency of self-microemulsifacation is shown in Table 8. After being diluted 250 times by different media, the particle diameters of GTP-PC-SNEDDS, from large to small, were: pH 6.8 PBS > 0.1 M HCl ≈ distilled water. It might be due to the NaCl in the PBS, which can cause salting out to lower the surfactant content and reduce the solubility of the surfactant, resulting in an increase in particle size. After repeated verification, the particle size in PBS was about two times larger than that in HCl, and the system was relatively stable. Considering the cost and ease of operation, the 0.1 M HCl was used to replace pH 6.8 PBS for the following investigation.

As described in Table 9, there was not a significant effect on the particle size of GTP-PC-SNEDDS when it was diluted 100–500 times by 0.1 M HCl. However, the particle size was remarkably reduced when it was diluted 1000 times, which might be due to the fact that SNEDDS was in a solution state after being infinitely diluted. In keeping with the previous investigations, the dilution factor was chosen to be 250 times.

In summary, the preparation process with the following parameters had the highest efficiency of self-microemulsifacation of GTP-PC-SNEDDS: the ratio of GTP-PC to blank was 1:10, and then the mixture was stirred at room temperature to form GTP-PC-SNEDDS, which was diluted 250 times by 0.1 M HCl, subsequently.

After many validation tests, the formulation with the most stable system was determined, and it had the highest solubility, a suitable particle size (<100 nm), and a shorter emulsion time (<2 min). Therefore, the optimal prescription was as follows: Maisin 35-1:Miglycol = 30%, Labrasol: Cremophor EL = 14 = 40%, Transcutol P = 30%, and Maisin 35-1:Miglycol = 12, and the ratio of GTP-PC to blank was 1:10; then, the mixture was stirred at 37 °C for 1 d and placed for 2 d to form stable GTP-PC-SNEDDS.

### 2.4. Characterization of GTP-PC-SNEDDS

#### 2.4.1. Transmittance Electron Microscope

The diluted GTP-PC-SNEDDS was examined by a transmission electron microscope. Because the particle size of GTP-PC-SNEDDS was only about 20 nm, which could easily form a relatively uniform spherical emulsion, the emulsion droplets were small and evenly dispersed under transmission electron microscope after negative staining with 1% phosphotungstic acid.

#### 2.4.2. Droplet Size and Zeta Potential

**Figure 5:**
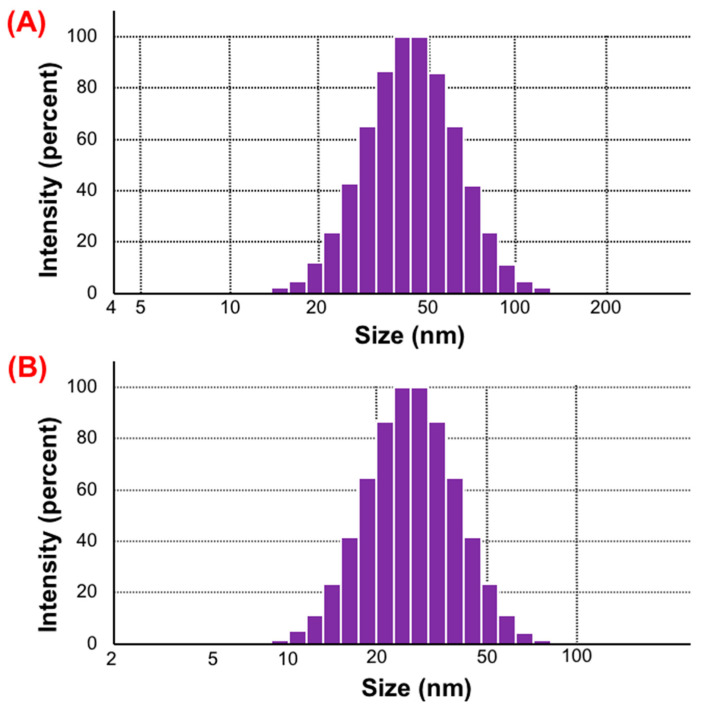
Characterization of GTP-PC-SNEDDS. (**A**): Particle Size distribution in dynamic light scattering (DLS) of SNEDDS at the dilution of 1:250 by pH 6.8 PBS. (**B**): Particle Size distribution DLS of SNEDDS at the dilution of 1:250 by pH 1.0 HCl.

As presented in Figure 5A,B, the average droplet sizes of GTP-PC-SNEDDS diluted by pH 1.0 HCl or pH 6.8 PBS were (25.2 ± 5.3) nm and (46.7 ± 8.2) nm, respectively, showing Gaussian distribution. The Zeta potentials of the GTP-PC-SNEDDS diluted by pH 1.0 HCl or pH 6.8 PBS were (−19.23 ± 5.84) mV and (−23.43 ± 3.93) mV, respectively, showing no significant difference (*p* > 0.05).

#### 2.4.3. Stability of Microemulsion Particle Size after Microemulsification

As described in Table 10, the particle size of the self-microemulsifying solution was not significantly changed within 8 h after dilution with 0.1 M HCl and distilled water (*p* > 0.05), and there was no drug for crystallization. However, the particle size of GTP-PC-SNEDDS diluted in pH 6.8 PBS significantly increased with time, and this might be due to the present of salt in PBS, which can reduce the surfactant content by salting out. The decrease of the surfactant content will reduce the surfactant solubilizing ability and weaken the self-microemulsifying ability, resulting in an increase in particle size. The larger particle size may be also due to the easily aggregation of the particles when dispersed in pH 6.8 PBS, with time increasing [39].

Therefore, since various salts are present in the intestinal tract, the particle size of the self-microemulsifying liquid in the intestinal tract easily became larger.

### 2.5. Bioavailability Experiments in Rats

**Figure 6:**
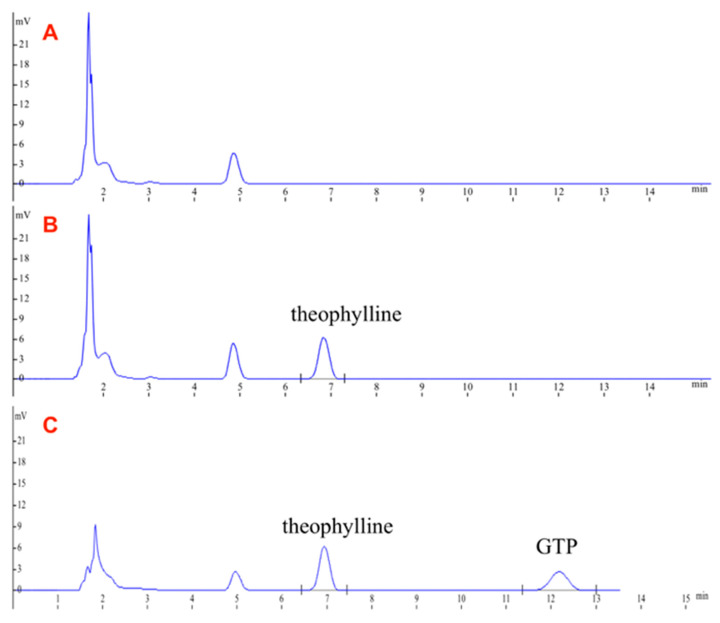
Typical chromatograms of (**A**) blank rat plasma; (**B**) blank rat plasma with theophylline; (**C**) blank rat plasma with theophylline and GTP.

Figure 6 shows the complete separation of the GTP in plasma under analytical conditions (A = 0.0133 C + 0.004, r = 0.9996), where C is the concentration ratio of GTP to theophylline, and A is the corresponding peak-area ratio of GTP/theophylline. The results attained from the RSD inter-days of low, middle, and high concentrations were 8.45%, 2.39%, and 3.78%, respectively. The corresponding RSD intra-days were 7.99%, 2.59%, and 5.42%, and the recoveries were (96.67 ± 6.22)%, (102.24 ± 2.71)%, and (102.40 ± 5.58)%, which indicated recoveries and RSD inter-days, and the RSD intra-days were satisfying, with the lowest detection limit at 50 ng·mL^−1^.

**Figure 7:**
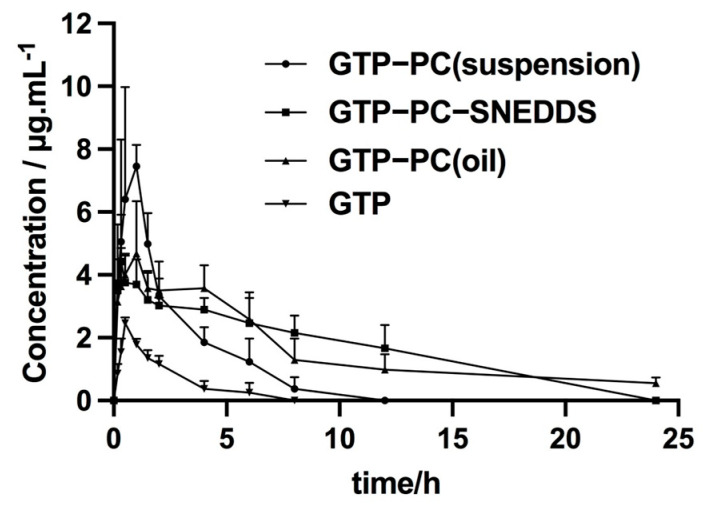
Mean plasma concentration–time curve of GTP, GTP-PC (suspension), GTP-PC (oil) solution, and GTP-PC-SNEDDS after single po dose of 80 mg/kg GTP in SD rats, n = 6.

Figure 7 and Table 11 illustrate the mean plasma concentration–time curve of GTP in rats after oral administration of GTP, GTP-PC, and GTP-PC-SNEDDS (equivalent to 80 mg/kg of GTP). All of them followed a single-compartment absorption model with first order. The main pharmacokinetic parameters of GTP and the other three formulations are presented in Table 11. As shown in Table 11, the C_max_, AUC_0–∞_, and Fr of three formulations were significantly higher than that of GTP, demonstrating that GTP was metabolized rapidly, and its higher bioavailability could be achieved by the formation of GTP-PC and GTP-PC-SNEDDS. Among the three formations, the bioavailability of GTP-PC-SNEDDS was highest, with approximately 2.6-fold and 1.3-fold of Fr value, compared with GTP-PC (suspension) and GTP-PC (oil solution), respectively. The improved bioavailability by GTP-PC and GTP-PC-SNEDDS might be attributed to the following reasons: (1) phospholipids are an important component of cell membrane, having the effects of keeping the cell membrane fluidity. Moreover, phosphatidylcholine and its digestion product lysophosphatidylcholine could promote lymphatic transport efficiently [40,41,42]. (2) Compared with that of GTP, the lipophilicity of GTP-PC was effectively increased, and the dissolution rate of GTP in GTP-PC was reduced. Therefore, improved bioavailability could be achieved by the use of delivery systems, which could enhance the rate and/or the extent of drug absorbing into intestinal mucosa [43]. (3) The instability of GTP in GI tract might decrease the bioavailability and activity of the GTP; however, the GTP-PC and GTP-PC-SNEDDS might protect it from metabolization by gastric secretions and gut bacteria [44,45,46] and improve the absorption of GTP. (4) By enhancing the solubility of bile to GTP, liver targeting may be facilitated, and phosphatidylcholine also acts as a hepatoprotective, hence giving the synergistic effect. Additionally, compared with GTP-PC, these fine droplets, with nano-size, produced by GTP-PC-SNEDDS could further enhance the dispersion of drug dissolved inside the oil phase into gastrointestinal fluid, resulting in the significant improvement of absorption in gastrointestinal (GI) tract.

## 3. Materials and Methods

### 3.1. Material and Animals

National Institute for Control of Pharmaceuticals supplied the reference substances of GTP with purity of 98%. GTP extract with a purity of 85% (*w*/*w*) was isolated in our laboratory and used for preparation of GTP-PC and GTP-PC-SNEDDS. Soybean phospholipids were obtained from Shanghai Tai-Wei pharmaceutical Co. Ltd. (Shanghai, China), and the phosphatidylcholine content was about 98% (*w/w*). Egg yolk phospholipid and hydrogenated phospholipid were presented by Lipoid Company (Ludwigshafen, LS, Germany). Imwitor 742 and Migyol 812N were presented by SASOL Company (Johannesburg, JB, South Africa). Maisin 35-1, Labrasol, Plurol Qleique CC 497, and Transcutol P were presented by GATTEFOSSE SAS Company (SAINT-PRIEST, SP, France). Cremophor EL was purchased from Shanghai Licheng Food Industry Co., Ltd (Shanghai, China). HPLC-grade methanol was purchased from TEDIA company, Inc. (Fairfield, CA, USA). A Milli-Q water purification system (Millipore, Bedford, MA, USA) was used to purify the water. All other chemicals were of analytical grade.

Male SD rats (180 ± 20 g) were supplied by Experimental Animal Center of Chinese Academy of Sciences. The rats were housed in an environmentally controlled room. Unless otherwise indicated, standard laboratory food and water were given. The Animal Experimentation Ethics Committee of Fudan University recommended all animal experimentation procedures (2019-03-YJ-WJX-01).

### 3.2. Chromatography

The GTP in GTP-PC was analyzed by LC-20AB HPLC system with SPD-20A UV detector (SHIMADZU, Kyoto, Japan) at 270 nm. The chromatography was performed on a Venusil MP C_18_ column 300A (150 mm × 4.6 mm, 5 μm) by using a mixture of methanol–water (30:70, *v/v*) as mobile phase at 1 mL/min and 30 °C. The GTP concentration in plasma samples were also detected following this method, except for the mobile phase, which was changed to a mixture of methanol–water (24:76, *v/v*).

The GTP content in GTP-PC was determined according to the external standard method. The peak area of GTP had a good linear relationship, with a concentration ranging from 19.84 to 744 μg/mL. The standard curve equation between peak area of GTP (A) and its concentration (C, μg/mL) was A = 6808.5C + (r = 0.9999). While the GTP was quantitatively determined by internal standard method, theophylline was used as internal standard. The linear regression curve between GTP concentration (C, μg/mL) and peak radio between GTP and theophylline (A) was calculated. The r value was 0.9996, indicating this curve had a good linear relationship (A = 0.033 C + 0.004).

### 3.3. Preparation of GTP-PC

#### 3.3.1. Complexation Efficiency of GTP-PC

The complexation efficiency of GTP-PC was measured as previously reported [36]. Briefly, methanol and dichloromethane were used to dissolve two samples of GTP-PC with approximately the same amount. After the dichloromethane solution was filtered, the GTP contents in both the methanol and dichloromethane solutions were determined by HPLC, as described in Section 3.2. The GTP in dichloromethane and methnol was regarded as complexed GTP and total GTP in GTP-PC (complexed and uncomplexed), respectively, because methanol can easily dissolve complexed and uncomplexed GTP in GTP-PC, while only complexed GTP in GTP-PC is dissolvable in dichloromethane. Therefore, the complexation efficiency was calculated as follows:

Complexation efficiency % = Wc/Wt × 100%

where Wc is the GTP content dissolved in dichloromethane, and Wt is the GTP content in methanol.

#### 3.3.2. Measurement of Dissociation Rate of GTP-PC

GTP was easily dissolved in water, but GTP-PC was practically insoluble. GTP dissolved in water was considered to be from the dissociation of GTP-PC. The dissolution studies could be carried out using the paddle method to assess the dissociation of GTP-PC [47]. At 37 °C, 200 mL of pH 6.8 phosphate buffer saline (PBS) was continually stirred at 50 rpm in dissolution flasks. The stirred medium was first containing GTP-PC (0.2 g). At 10, 20, 30, 45, 60, and 120 min, 5 mL samples were withdrawn and filtrated with 0.45 μm cellulose nitrate membranes, and then 5 mL fresh mediums were added into flask. The GTP concentration in the resulting solution was determined by HPLC by mixing 0.4 mL filtrate with 4 mL PBS. The dissociation rate was calculated according to the following equation [48]:

Log C = Log C_0_ − Kt/2.303

where C_0_ is the initial concentration of GTP, K is the first order rate constant, and t is the time in hours.

#### 3.3.3. Investigations of GTP-PC

GTP and phospholipid were placed in a round-bottom flask and suspended in different solvents (trichloromethane, ethyl acetate, and tetrahydrofuran) with a certain molar ratio (GTP: phospholipids = 2:1, 1:1, 1:2, 1:3, and 1:4). Then, the mixture was stirred at 40 °C for different times (0.5 h, 1 h, 2 h, 3 h, 4 h, 5 h, and 6 h). The dried residues were collected as GTP-PC after the solvent was evaporated off under vacuum at 40 °C. By measuring complexation efficiency and dissociation, the optimal preparation process for GTP-PC was determined.

### 3.4. Characterization of GTP-PC

#### 3.4.1. Differential Scanning Calorimetry (DSC)

In a nitrogen atmosphere, samples sealed in aluminum crimp cells were heated at 10 °C/min from 0 °C to 300 °C (PERKIN-ELMER 7, Waltham, MA, USA). The peak transition onset temperatures of four types of samples were compared, including phospholipid, GTP, the mixture of phospholipid and GTP, and the GTP-PC.

#### 3.4.2. X-ray Diffractometry (XRD)

Using a graphite monochromator with Cu/Ka radiation with a voltage window of 40 kV and current density of 60 mA with a scanning rate of 4 °C/min ranging from 5 °C to 45 °C, the X-ray diffraction (D/MAXX Rigaku, Tokyo, Japan) was performed.

#### 3.4.3. Fourier Transform Infrared Spectra (FT-IR)

Samples were compressed into a KBr pellet, and their FT-IR spectra were recorded by FT-IR spectrometer (Avatar TM 360E.S.P.TM, Avatar).

#### 3.4.4. Thin-Layer Chromatography (TLC)

Sample was prepared by dissolving standard GTP, GTP-PC, the physical mixture, and GTP-PC in methanol. The experiment was carried out according to a TLC test of China pharmacopoeia. A total of 5 μL solution was spotted onto a silica gel GF254 plate with ethyl acetate-menthol-water (20:2:1, *v/v/v*) as developing solvent. The resulting plate had a picture taken under ultraviolet lamp (254 nm).

#### 3.4.5. Ultraviolet (UV) Spectra

Sample methanol solutions were scanned by a UV spectrometer over the wavenumber range of 200–400 nm.

#### 3.4.6. Dissolution Studies in Phosphate Buffer Saline (pH 6.8)

The dissolution studies were carried out according to paddle method demonstrated in Section 3.3.2. Additionally, samples were withdrawn at 10 min, 20 min, 30 min, 1 h, 2 h, 4 h, 6 h, 8 h, and 12 h.

### 3.5. Preparation of GTP-PC-SNEDDS

#### 3.5.1. Solubility Studies

Suitable excipients for preparation of GTP-PC were screened by solubility studies. Briefly, excess GTP-PC was added into about 1 g of various oils, surfactants, or co-surfactants, respectively. Then, the mixture was shaken at 37 °C for 48 h; after centrifuging at rpm for 10 min, the content of GTP was assayed by HPLC, and the GTP solubility of GTP-PC in each oily medium was calculated.

#### 3.5.2. Construction of Pseudo-Ternary Phase Diagrams

Pseudo-ternary phase diagrams can be used for preliminary screening of self-microemulsifying systems. The points in the triangle represent the different composition ratios of ternary systems, such as oil phase, surfactant, and cosurfactant. In this work, about 1 g of blank formulation (only including different ratios of oil phase, surfactant, and cosurfactant) was first weighed accurately, and then about 10% (*w/w*) of GTP-PC was added. The mixture was stirred at 37 °C for 24 h and followed to keep for 48 h to observe whether all the drugs were dissolved and formed a uniform and transparent solution. If so, part of formulation (equivalent to 4 mg of GTP) was diluted 250 times with 0.1 M HCl and then stirred at 37 °C and 50 rpm. If a clear and transparent solution could be formed, it is believed that the formulation, including this ratio of oil phase, surfactant, and cosurfactant, could been used to form microemulsion and draw pseudo-ternary phase diagrams, in turn.

#### 3.5.3. Investigations of GTP-PC-SNEDDS

In order to optimize the preparation process of GTP-PC-SNEDDS, the effects of weight ratios of GTP-PC to blank SNEDDS, preparation technology, dilution media, and dilution multi were further investigated.

#### 3.5.4. Characterization of GTP-PC-SNEDDS

##### Morphological Characterization

Transmission electron microscope (TEM) (PHILIPS CM-120, Eindhoven, EIN, Netherlands) was used to observe the morphology of SMEDDS. SMEDDS was diluted with distilled water 1:25 and gently shaken to mix. Afterwards, a drop of the diluted sample was placed on copper grids, and the excess was rubbed off with filter paper. The grids were then stained with 1% phosphor-tungstic acid solution for 30 s.

##### Droplet Size and Zeta Potential

Droplet size distribution and zeta potential of GTP-PC-SNEDDS were determined using NICOMP 380 ZLS Zeta Potential/Particle Sizer (PSS-380, NICOMP, Santa Barbara, CA, USA).

##### Solubility

Excess GTP-PC was added into about 0.5 g of blank formulation, which was shaken at 37 °C for 48 h and then centrifuged at 15,000 rpm for 10 min. The solubility of GTP in blank formulation was evaluated by the content measurement of GTP in supernatant by HPLC.

##### Self-Microemulsifying Time

The self-microemulsifying time of GTP-PC-SNEDDS was analyzed according to Chinese pharmacopoeia (2015 edition). Certain amount of formulation (equivalent to 4 mg of GTP) was diluted 250 times with 0.1 M HCl, and then stirred at 37 °C and 50 rpm. Self-microemulsifying time was recorded, since the droplets contacted with the liquid level to a clear and transparent solution was formed. According to the literature, the time of self-microemulsification should not exceed 2 min.

### 3.6. Bioavailability Experiments in Rats

#### 3.6.1. Plasma Sample Preparation and Validity

A total of 250 μL internal standard solution (4.031 μg/mL theophylline in methanol solution) was added to 100 μL plasma and agitated for 30 s. Then, the solution was centrifuged (15 min). Aliquots (20 μL) of the supernatant were injected for HPLC analysis.

Rat plasma blanks were supplemented with different amounts of GTP for validation of the method. The resulting concentrations of GTP were 0.13, 0.53, 5.3, 21.2, and 42.4 μg/mL. For testing the method’s precision, accuracy, and detection limit, the calibrations were subjected to the above analytical procedure.

#### 3.6.2. Pharmacokinetic Study

Twelve four male rats (150–200 g) were divided randomly into four groups, and each group had six animals. They were fasted for 24 h, but allowed to take water freely. They were orally administered GTP solution in water (equivalent to 80 mg/kg of GTP), GTP-PC suspension in oil (equivalent to 80 mg/kg of GTP), GTP-PC suspension in water (equivalent to 80 mg/kg of GTP), and GTP-PC–SNEDDS (equivalent to 80 mg/kg of GTP), respectively. About 0.4 mL blood samples were collected from the tail vein into tubes containing heparin at 0.17, 0.33, 0.5, 1, 1.5, 2, 4, 6, 8, 12, and 24 h. Plasma was separated by centrifugation (5000 rpm, 10 min) and stored at −20 °C until analysis.

Peak concentration (C_max_) and peak times (t_max_) were derived directly from the experiment points, and AUC_0–∞_ was calculated by trapezoidal method. A computer program called 3p87 was used to compute the rest of the pharmacokinetic parameters.

## 4. Conclusions

In this study, we firstly prepared GTP-PC by studying the factors affecting the formation of GTP-PC with complexation efficiency and a dissociation experiment. DSC, XRD, FT-IR, TLC, and UV measurements confirmed that GTP is bonded to phospholipids only through hydrogen bonds and/or van der Waals forces. Then, GTP-PC was loaded into SNEDDS by investigating the effects of the weight ratios of GTP-PC to blank SNEDDS, preparation technology, dilution media, and dilution multi based on the screening results of the oils, surfactants, and cosurfactants. As a result, the dissolution of the GTP in GTP-PC was delayed, compared with GTP material. In rats, GTP-PC and GTP-SNEDDS could significantly enhance GTP bioavailability, compared with GTP. Our study may serve as a basis for the clinical applications of GTP-PC and GTP-PC-SNEDDS.

There are also the same limits for this work, for example, the morphology and sizes of aggregates of GTP-PC were not characterized. The characterization of SNEDDS without GTP-PC, including size, Z-potential, and PDI, was not clarified. Furthermore, several studies need to be further executed, such as regarding the absorbed mechanism of GTP-PC and GTP-PC-SNEDDS, an experiment with the delivery of GTP to show that the GTP-PC and GTP-PC-SNEDDS are really able to work in vivo.

## Figures and Tables

**Table 1:** Complexation efficiency and dissociation rate of GTP-PC of different types of phospholipids.

Type of Phospholipids	Complexation Efficiency (%)	K (h^−1^)
soybean phospholipid	97.15 ± 3.27	1.60
egg yolk phospholipid	100 ± 4.92	2.49
hydrogenated phospholipid	88.20 ± 4.74	8.52

**Table 2:** Dissolubility of GTP, GTP-PC, and phospholipids in different solvents (mg/mL).

Solvents	Soybean Phospholipid	GTP	GTP-PC
Water	※	++	※
Methanol	+	++	+
Ethanol	－	－	※
Acetone	++	－	－
Dichloromethane	++	－	++
Tetrahydrofuran	++	－	++
Ethyl Acetate	++	－	++
Trichloromethane	++	+	++
Ethyl Ether	－	－	－
n-hexane	++	－	－

－ indicates substance is not soluble in the solvent within 48 h; + indicates substance is soluble in the solvent within 24 h; ++ indicates substance is easily dissoluble in the solvent within 8~12 h; ※ indicates substance is dispersed in the solvent within 48 h.

**Table 3:** Complexation efficiency and dissociation rate of GTP-PC of different types of solvent.

Type of Solvent	Complexation Efficiency (%)	K (h^−1^)
trichloromethane	61.71 ± 5.38	3.17
tetrahydrofuran	97.15 ± 3.31	1.99
ethyl acetate	86.77 ± 4.24	2.53

**Table 4:** Complexation efficiency and dissociation rate of phytosome of different molar ratios of GTP and phospholipid.

Molar Ratio of GTP and Phospholipids	Complexation Efficiency (%)	K (h^−1^)
2:1	86.82 ± 6.53	5.21
1:1	97.15 ± 3.31	3.72
1:2	99.87 ± 3.21	2.39
1:3	99.66 ± 1.14	0.72
1:4	99.79 ± 2.23	0.55

**Table 5:** The solubility of GTP-PC in various vehicles at 37 °C (n = 3, g/g).

Vehicle	Solubility of GTP-PC (g/g)
Oils	
Imwitor742	0.146 ± 0.028
Maisin 35-1	0.037 ± 0.016
Miglycol 812N	-
Maisin 35-1:Miglycol 812N = 1:2	0.095 ± 0.016
Maisin 35-1:Miglycol 812N = 1:1	0.105 ± 0.013
Maisin 35-1:Miglycol 812N = 2:1	0.065 ± 0.009
Labrafill M 1994 CS	0.074 ± 0.019
Imwitor742:Miglycol 812N = 2:1	0.127 ± 0.013
Imwitor742:Miglycol 812N = 1:2	0.132 ± 0.011
Imwitor742:Miglycol 812N = 1:1	0.138 ± 0.038
Plurol Qleique CC 497	0.016 ± 0.006
Surfactant	
Labrasol	0.107 ± 0.011
Cremophor EL	0.025 ± 0.005
Labrasol:Cremophor EL = 1:1	0.058 ± 0.005
Labrasol:Cremophor EL = 1:2	0.070 ± 0.009
Labrasol:Cremophor EL = 1:3	0.069 ± 0.014
Labrasol:Cremophor EL = 1:4	0.060 ± 0.009
Tween 80	0.012 ± 0.004
Co-surfactant	
Transcutol P	0.315 ± 0.012
PEG400	-
Absolute ethanol	-
95% ethanol	-

**Table 6:** The particle size of GTP-PC-SNEDDS with different ratios of drug/blank formula (*w*/*w*) in 0.1 M HCl, pH 6.8 PBS, and water (nm).

GTP-PC/Blank Formula (*w*/*w*)	0.1 M HCl Particle Size (nm)	pH 6.8 PBS Particle Size (nm)	Water Particle Size (nm)
1:5	190.0 ± 23.5	527.5 ± 34.7	205.6 ± 38.1
1:7	47.5 ± 15.2	150.1 ± 21.1	44.6 ± 12.8
1:10	27.8 ± 3.9	52.7 ± 8.4	24.6 ± 3.4

**Table 7:** The effect of preparation technology on the efficiency of self-microemulsifacation of GTP-PC-SNEDDS.

Preparation Technology	Particle Size (nm)	Time (s)	Appearance	Appearance after Dilution
Ultrasonic	31.2 ± 0.6	69 ± 15	Clear liquid	Turbid, clear after 30 min
Stirring (at room temperature, 25 °C)	27.8 ± 3.9	21 ± 6	Clear liquid	Clear liquid
Volution	32.6 ± 3.0	47 ± 12	Clear liquid	Turbid, clear after 30 min
Placed at 4 °C	26.2 ± 2.0	32 ± 9	Layering	Clear liquid
Placed at 25 °C	31.7 ± 2.4	26 ± 5	Layering	Clear liquid
Placed at 30 °C	31.3 ± 0.4	23 ± 7	Layering	Clear liquid
Placed at 40 °C	27.7 ± 0.1	29 ± 3	Layering	Clear liquid

**Table 8:** The effects of different dilution media on the efficiency of self-microemulsifacation of GTP-PC-SNEDDS.

Different Media	Particle Size (nm)
0.1 M HCl	27.8 ± 3.9
pH 6.8 PBS	46.7 ± 2.1
Distilled Water	24.6 ± 3.4

**Table 9:** The effects of different dilution multi on the efficiency of self-microemulsifacation of GTP-PC-SNEDDS.

Diluted Multi	Particle Size (nm)
100	29.7 ± 4.4
250	27.8 ± 3.9
500	27.5 ± 4.5
1000	3.2 ± 0.6

**Table 10:** Stability of particle size after self-microemulsifying in 0.1 M HCl, pH 6.8 PBS, and distilled water at 37 °C.

Time (h)	HCl Particle Size (nm)	pH 6.8 PBS Particle Size (nm)	Distilled Water Particle Size (nm)
0	27.8 ± 3.9	46.7 ± 2.1	24.6 ± 3.4
1	30.5 ± 5.0	56.7 ± 8.5	28.2 ± 4.1
2	31.5 ± 4.4	62.7 ± 8.4	30.8 ± 4.8
4	32.7 ± 4.3	61.6 ± 5.7	31.4 ± 3.9
8	35.4 ± 5.7	65.3 ± 6.2	34.6 ± 2.5

**Table 11:** Pharmacokinetic parameters and bioavailability of GTP, GTP-PC (suspension), and GTP-PC (oil solution) to SD rats (n = 6).

Parameter	GTP	GTP: Phospholipids = 1:2 (Suspension)	GTP: Phospholipids = 1:2 (Oil Solution)	GTP-PCSMEDDS
A	3.486 ± 1.391	8.961 ± 2.677	4.666 ± 0.904	3.954 ± 0.554
K_a_/h^−1^	5.362 ± 3.319	2.275 ± 0.411	6.303 ± 3.873	18.123 ± 15.921
K_e_/h^−1^	0.550 ± 0.295	0.338 ± 0.059	0.115 ± 0.038	0.086 ± 0.045
T_1/2Ka_/h	0.160 ± 0.099	0.310 ± 0.056	0.110 ± 0.061	0.061 ± 0.042
T_1/2Ke_/h	1.473 ± 0.790	2.085 ± 0.364	6.049 ± 1.351	10.233 ± 6.400
T_max_/h	0.604 ± 0.138	1.001 ± 0.070 *	0.665 ± 0.388	0.452 ± 0.306 *
C_max_/μg·mL^−1^	2.417 ± 0.227	7.365 ± 0.760 **	4.682 ± 1.645 *	4.369 ± 1.503 *
AUC_0–∞_/μg·mL^−1^·h	6.273 ± 2.123	23.375 ± 10.665 **	47.009 ± 20.532 **	60.749 ± 33.759 **
Fr (%)	-	372.65	749.45	968.49

* *p* < 0.05 and ** *p* < 0.01 indicate a statistically significant difference, when compared with GTP concentration used Student’s *t* test; K_a_: rate constant of absorption; K_e_: rate constant of elimination; T_1/2Ka_: absorption half-life; T_1/2Ke_: elimination half-life; T_max_: time to reach peak concentration; C_max_: the maximum concentration; AUC_0–∞_: the area under the plasma concentration–time curve; Fr: relative bioavailability.

## Data Availability

Data is contained within the article.
